# The transmission potential of malaria-infected mosquitoes (*An.gambiae-*Keele, *An.arabiensis-*Ifakara) is altered by the vertebrate blood type they consume during parasite development

**DOI:** 10.1038/srep40520

**Published:** 2017-01-17

**Authors:** S. Noushin Emami, Lisa C. Ranford-Cartwright, Heather M. Ferguson

**Affiliations:** 1 Institute of Infection, Immunity and Inflammation, College of Medical, Veterinary & Life Sciences, University of Glasgow, Glasgow, Scotland, UK; 2 Institute of Biodiversity, Animal Health and Comparative Medicine, College of Medical, Veterinary & Life Sciences, University of Glasgow, Glasgow, Scotland, UK

## Abstract

The efficiency of malaria parasite development within mosquito vectors (sporogony) is a critical determinant of transmission. Sporogony is thought to be controlled by environmental conditions and mosquito/parasite genetic factors, with minimal contribution from mosquito behaviour during the period of parasite development. We tested this assumption by investigating whether successful sporogony of *Plasmodium falciparum* parasites through to human-infectious transmission stages is influenced by the host species upon which infected mosquitoes feed. Studies were conducted on two major African vector species that generally are found to differ in their innate host preferences: *Anopheles arabiensis* and *An. gambiae sensu stricto*. We show that the proportion of vectors developing transmissible infections (sporozoites) was influenced by the source of host blood consumed during sporogony. The direction of this effect was associated with the innate host preference of vectors: higher sporozoite prevalences were generated in the usually human-specialist *An. gambiae s.s.* feeding on human compared to cow blood, whereas the more zoophilic *An. arabiensis* had significantly higher prevalences after feeding on cow blood. The potential epidemiological implications of these results are discussed.

Malaria is the most important vector-borne disease affecting humans, with approximately 3.2 billion people exposed globally in 2015^1^. Malaria is caused by protozoan parasites (*Plasmodium* species) transmitted by *Anopheles* mosquitoes. Over 90% of malaria cases and deaths are due to *Plasmodium falciparum*, and occur in sub-Saharan Africa^1^, where the major vectors are mosquitoes of the *Anopheles gambiae sensu lato (s.l.*) complex (including *An. gambiae sensu stricto (s.s.*), *An. coluzzii*, and *An. arabiensis)* and *An. funestus*. Over the last 15 years the prevalence of *P. falciparum* has fallen by ~40% across Africa, primarily due to the widespread use of Long Lasting Insecticide Nets (LLINs)^2^. This control method targets vector species that feed on people, indoors, and at night^3^. LLIN coverage has been associated with shifts in vector species composition^4^,^5^,^6^, with more substantial declines observed for vector species that feed primarily on people and indoors (e.g. *An. gambiae s.s.* and *An. coluzzii*^7^,^8^,^9^). Prior to 2013^10^, *An. gambiae* s.s. and *An. coluzzii* were considered to be the same species (known as *An. gambiae* s.s.) with almost all published accounts of its host choice indicating a strong preference for human hosts^7^,^9^,^11^,^12^,^13^,^14^,^15^,^16^. In contrast, although *An. arabiensis* regularly feeds on humans and cattle, it is frequently reported to have a preference for cattle when both host types are available^7^,^9^,^14^,^15^,^16^,^17^,^18^,^19^,^20^,^21^,^22^,^23^,^24^,^25^,^26^,^27^,^28^. Whilst there is some geographic variation in the host feeding patterns of these vector species (e.g. an *An. gambiae s.s.* population which prefers feeding on dogs^29^, an *An. arabiensis* population that feeds exclusively on people^28^), overall the general trend of a high preference for humans by *An. gambiae s.s.* and *An. coluzzii*, and for cattle by *An. arabiensis*, holds across a broad range of settings. Consequently, the epidemiological consequences of shifts in vector species may be complex, as they will influence both how frequently humans are bitten by mosquitoes, and how effectively mosquitoes are targeted by control measures.

Variation in feeding behaviour within these malaria vectors has potential to influence malaria transmission in another way: the type of host blood consumed by infected mosquitoes could influence the quality and quantity of resources available to developing parasites, and thus their likelihood of onward transmission. After ingestion by mosquitoes, malaria parasites require at least 10–14 days of further development within vectors (sporogony) before they can be transmitted to a new host^30^. During this period mosquitoes will still blood feed every 2–4 days in accordance with their regular gonotrophic cycle^31^,^32^. The type of host blood consumed by infected mosquitoes (e.g. human or bovine) could influence parasite development in several ways. Malaria parasites require energetic resources from blood to fuel their development in mosquitoes^33^. Haematological properties such as biochemical composition, and red cell density and size vary between vertebrate species^34^,^35^, and may affect the nutritional value of host blood to mosquito-stage *Plasmodium. Anopheles* may have adapted to optimize extraction of resources from the blood of preferred host species, resulting in enhanced longevity^36^, which indirectly benefits parasites by ensuring vectors survive long enough for them to complete sporogony. The differential blood feeding behaviour of *An. gambiae s.s* and *An. arabiensis* means that malaria parasites developing in these vectors will be exposed to different blood sources. Prediction of the epidemiological consequences of shifts in vector species composition will require an understanding of both how vectors differ in their innate susceptibility to infection, and how their subsequent behaviour and fitness during sporogony influences their likelihood of developing transmission stage parasites. To date, the potential impact of mosquito host choice on malaria parasite sporogony is unknown.

This study addressed these knowledge gaps by investigating if: (1) the host species from which malaria-infected mosquitoes feed (human or cattle) influences parasite sporogonic success; and (2) the efficiency of *P. falciparum* malaria parasite sporogony varies between *An. arabiensis* (Ifakara) and *An. gambiae s.s.* (Keele). Assessing the contribution of mosquito host choice and vector species to parasite sporogony has particular relevance for understanding the potential epidemiological consequences of recently reported changes in the ecology of African malaria vectors.

## Results

For brevity in this section, results obtained with our colonies of *An. gambiae s.s.* (Keele) and *An. arabiensis* (Ifakara) are referred to by species name only.

### Oocyst prevalence

**Figure 1:**
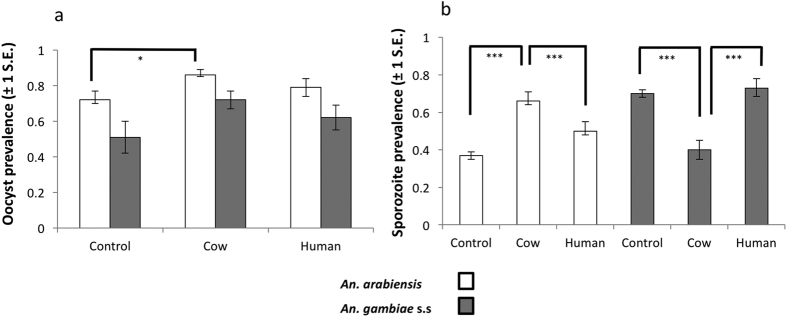
The prevalence of *P. falciparum* oocysts and sporozoites in relation to the source of host blood they consumed 4 days after infection. (**a**) Oocyst and (**b**) sporozoite prevalence was measured on day 10 and 16 post infection respectively. The infection prevalence values are taken from the logistic regression model estimations and the error bars are standard errors. White bars show *An. arabiensis* (Ifakara), and gray bars are for *An. gambiae s.s.* (Keele). Statistically different comparisons are shown by the brackets (***p ≤ 0.001; *p = 0.01).

Of the 1440 mosquitoes fed on malaria-infected blood (720 for each vector species; Supplementary Table S1), the proportion infected with oocysts (prevalence) across replicates ranged from 71–86% in *An. arabiensis,* and 54–71% in *An. gambiae s.s.* (Fig. 1a). Overall, prevalences were significantly higher in *An. arabiensis* than *An. gambiae s.s.* (Fig. 1a, z = 3.14, p = 0.001). For *An. arabiensis*, oocyst prevalence was significantly higher in groups consuming cow blood 4 days after the infectious blood meal than in the control group having no second blood meal (Fig. 1a; z = 2.43, p = 0.01). There was no significant difference in oocyst prevalence between *An. arabiensis* that consumed human blood during sporogony and unfed controls (z = 1.19, p = 0.23). Within *An. gambiae s.s.*, oocyst prevalence was similar regardless of the type of blood consumed by infected mosquitoes (Fig. 1a; χ^2^_2_ = 0.16, p = 0.69). Oocyst prevalence was not significantly associated with body size in either mosquito species (χ^2^_1_ = 0.46, p = 0.49).

### Sporozoite prevalence

The proportion of mosquitoes that developed sporozoite infections (sporozoite prevalence) was generally higher in *An. gambiae s.s.* than *An. arabiensis* (Fig. 1b; z = 3.57, p < 0.001). However, sporozoite prevalence was significantly influenced by the interaction between mosquito species and the type of blood consumed during sporogony (Fig. 1b; χ^2^_1_ = 28.36, p < 0.001). *An. arabiensis* fed on cow blood had significantly higher sporozoite prevalence than those fed human blood (z = −3.41, p = 0.001), or the unfed controls (Fig. 1b; z = 3.99, p < 0.001). Sporozoite prevalence was similar in *An. arabiensis* fed on human blood and the unfed controls (z = 0.70, p = 0.76). By contrast, sporozoite prevalence was significantly lower in *An. gambiae s.s.* that consumed cow blood during sporogony, compared to those fed human blood (z = −3.94, p < 0.001), or the unfed controls (Fig. 1b; z = −3.55, p = 0.001). Sporozoite prevalence was similar for *An. gambiae s.s.* fed on human blood and the unfed controls (z = 0.36, p = 0.92). There was no association between mosquito body size and the probability of developing a sporozoite infection in either *Anopheles* species (χ^2^ = 1.59, p = 0.21).

### Development of oocyst to sporozoite infection

As described above, sporozoite prevalence was generally higher in *An. gambiae s.s.*, despite higher prevalence in *An. arabiensis* during the earlier oocyst stage. The proportion of *An. arabiensis* with parasite infection fell by 50% between oocyst and sporozoite stage, regardless of host blood type consumed during sporogony (p > 0.05 for all 2 way interactions). By contrast, the proportion of *An. gambiae s.s.* harbouring sporozoite infections was equal or higher than the proportion detected with oocyst infections in both the human blood fed and control group (human fed vs. control: z = 2.02, p = 0.07). However, infection prevalence fell markedly between oocyst and sporozoite stage for *An. gambiae s.s.* fed on cow blood (cow fed vs. control: z = −3.99, p < 0.001; cow fed vs. human: z = −3.78, p < 0.001, Fig. 1).

### Parasite loads in the midgut and salivary glands

**Figure 2:**
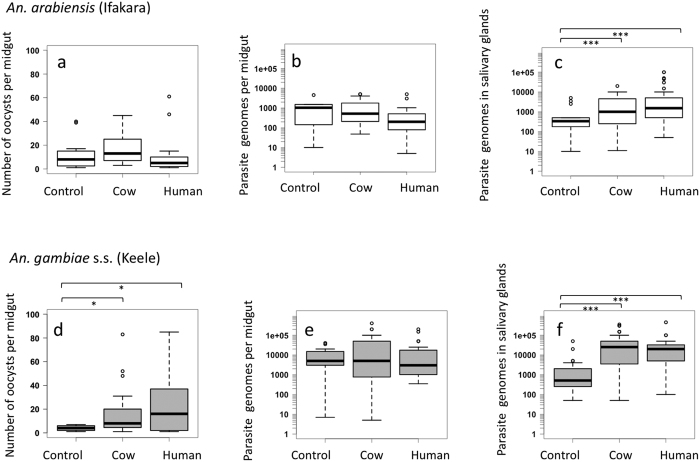
Effect of second blood meal from different hosts (cow and human) on oocyst and sporozoite load in mosquitoes. Four days after the infectious blood, *An. gambiae s.s.* (Keele) and *An. arabiensis* (Ifakara) mosquitoes were offered a second blood meal of human or cow origin, or no second blood meal (control). Number of oocysts per midgut (panels a and d) were measured at day 10 post-infection. The total number of parasites per mosquito was estimated using quantitative PCR within the midgut (oocyst) stages at day 10 post-infection (panels b and e) and within the salivary glands at day 16 post-infection (panels c and f). The top panels (**a,b,c**) show *An. arabiensis* (Ifakara), and the lower panels (**c,d,e**) show *An. gambiae s.s.* (Keele). The infection load values are taken from the negative binomial model estimations. The median is represented as a thick solid line, the box represents the upper and lower quartile range, and the whiskers show the range. Outliers are shown as unfilled circles. Statistically different comparisons are shown by the brackets (***p ≤ 0.001; *p = 0.01). Data are for infected mosquitoes only (for data on all mosquitoes, see Supplementary Table S2).

Overall, the number of oocysts per mosquito (parasite load) was higher in *An. arabiensis* than in *An. gambiae s.s.* (Fig. 2a,d; z = −3.22, p = 0.001). Oocyst loads in *An. arabiensis* were similar regardless of the type of host blood consumed during sporogony (Fig. 2a; deviance = 4.68, d.f. = 2, p = 0.19). However, oocyst loads in *An. gambiae s.s.* fed on any type of blood (human or cow) were higher than unfed controls (Fig. 2d; cow blood: z = 2.89, p = 0.01; human blood: z = 2.48, p = 0.03); but did not differ between those fed human or cow blood (z = −1.12, p = 0.10). There was no significant association of oocyst load and mosquito body size (deviance = 4.74, d.f. = 2, p = 0.09).

Although oocyst numbers in *An. arabiensis* were higher than in *An. gambiae*, the number of sporozoites developing within those oocysts at day 10 post infection was higher in *An. gambiae* than *An. arabiensis* (Fig. 2b,e; deviance = 12.96, d.f. = 1, p < 0.001). The type of host blood consumed during sporogony did not significantly influence the total number of parasites developing within oocysts in either mosquito species (*An. arabiensis*: deviance = 3.90, d.f. = 2, p = 0.14; *An. gambiae s.s.*: deviance = 3.38, d.f. = 2, p = 0.18). Neither oocyst numbers (deviance = 0.80, d.f. = 1, p = 0.37) nor mosquito body size (deviance = 0.18, d.f. = 1, p = 0.67) were significant predictors of the total number of parasites in oocysts.

The total number of sporozoites estimated to be in mosquito salivary glands (Fig. 2c,f) was higher in *An. gambiae s.s.* than in *An. arabiensis* (deviance = 35.92, d.f. = 1, p < 0.001). Mosquitoes that had any type of blood meal during sporogony (human or cow) developed more salivary gland sporozoites than unfed controls (*An. arabiensis*: deviance = 57.88, d.f. = 2, p < 0.001; *An. gambiae s.s.*: deviance = 80.36, d.f. = 2, p < 0.001), but there was no difference in sporozoite numbers between mosquitoes fed on human or cow blood (*An. arabiensis*: z = −1.13, p = 0.48; *An. gambiae s.s.*: z = −1.29, p = 0.38).

## Discussion

This study provides evidence that the source of blood consumed by mosquitoes carrying developing malaria parasites has a significant impact on the successful completion of the parasite sporogonic development. We demonstrate that the proportion of vectors developing transmission-stage sporozoites is significantly influenced by the type of host blood on which they feed during sporogony. As only mosquitoes that develop sporozoite infections are capable of transmitting malaria, we consider this outcome to be the most epidemiologically relevant, and a prime determinant of parasite fitness. Furthermore, by comparing colonies of two major African malaria vector species, we showed there was no single optimal host blood type for malaria parasite development. For *An. gambiae s.s.* (Keele), a species which usually feeds on humans, sporogonic success was lowest when consuming cow blood during sporogony. In contrast, sporogonic success was highest after consuming cow blood for the usually more zoophilic species, *An. arabiensis* (Ifakara). These host-specific impacts were not evident during earlier stages of sporogonic development (e.g. oocyst prevalence), and were not associated with any measure of parasite load. These findings could be the result of differential blood meal size, if mosquitoes take a larger blood meal from their preferred host. However, although not measured directly in these experiments, a previous study found no difference in average blood meal size taken from humans and cows for either vector species^9^.

Our results pertain to one colony of *An. arabiensis* (Ifakara) and one of *An. gambiae s.s.* (Keele). The host preference of these mosquito colonies was not reconfirmed during the period of study. The *An. arabiensis* colony used here was derived from a field population in southern Tanzania ~3 years before this study, and is known to have a strong preference for feeding on cattle; approximately 78% of blood meals were from cattle when humans and cattle were both available^37^. The *An. gambiae s.s.* strain used was derived from a mixture of four other lines that were colonised between 20 and 40 years ago^38^ (see Supplementary Information). Whilst the host preferences of these founder populations at the time of colonisation were not described, almost all other reports for this species indicate a consistent and clear preference for human hosts^7^,^9^. Thus, we hypothesise that the host species preferences of these mosquito colonies remain reflective of what would be expected in wild populations, but further work is needed to confirm this.

Additionally, a major caveat is that our results are specific to one colony of each species, and cannot be extrapolated to the whole species. Mosquitoes maintained in colonies for several generations can adapt to laboratory conditions, and it is useful to consider how this could have impacted our results. For practical reasons, mosquito colonies used here are maintained by membrane feeding on human blood, rather than direct feeding on hosts (e.g. humans or cows). The provision of human blood to *An. arabiensis* is not unnatural, as this vector regularly feeds on both humans and cattle in the wild. Their preference for cattle arises only when both humans and cattle are available, but when cattle are absent it feeds readily on humans^37^. However, it is possible that *An. arabiensis* in our colony became better adapted to human blood than a wild population that also have access to cattle. If this is the case, one might expect *An. arabiensis* to have a similar response to host blood variation as *An. gambiae*: e.g. parasite development is most efficient after feeding on human blood, and reduced when they feed on cattle. This was not what we observed: our *An. arabiensis* colony had a distinct response to cattle blood despite being maintained on human blood for 3–4 years. This does not discount the possibility that our results were influenced by laboratory adaptation, as perhaps the response to cow blood in *An. arabiensis* would be even more extreme in natural populations. Further investigation of in natural populations is encouraged to test this.

*An. arabiensis* (Ifakara) acquired a higher prevalence and intensity of oocysts, compared to that of *An. gambiae s.s.* (Keele), after feeding on the same blood containing infectious *P. falciparum* gametocytes. Despite developing more oocysts than *An. gambiae s.s.* (Keele), the resulting parasite loads in *An. arabiensis* (Ifakara) were lower in the midgut (parasites developing within oocysts) and salivary gland stages (sporozoites) than for *An. gambiae s.s.* (Keele). *An. arabiensis* (Ifakara) is therefore a less permissive host than *An. gambiae s.s.* (Keele) for the development of *P. falciparum*. Approximately half of the midgut infections in *An. arabiensis* (Ifakara) failed to develop into salivary gland sporozoites, resulting in a lower sporozoite prevalence in this species compared to *An. gambiae s.s.* (Keele). By contrast, the development of parasites from the midgut to salivary gland stage was highly efficient in *An. gambiae s.s.* (Keele), and despite acquiring a lower prevalence and intensity of oocyst infections, *An. gambiae s.s.* (Keele) developed a higher prevalence and number of salivary gland sporozoites, consistent with the greater contribution of this species to malaria transmission. The efficiency with which oocyst midgut infections progress to the infective sporozoite stages in the salivary glands is therefore significantly greater in *An. gambiae s.s.* (Keele) than *An. arabiensis* (Ifakara).

Additional blood meals during sporogony (of any blood type) did not change the successful development of oocysts to infective sporozoite stages in *An. arabiensis* (Ifakara), whereas additional blood feeds, and the source of the blood, significantly affected their development in *An. gambiae s.s.* (Keele). Consuming cow blood during sporogony significantly decreased parasite development compared to human blood, and even compared to no blood feeding at all. Since *An. gambiae s.s.* usually exhibits strong anthropophily in nature, this decrease in efficiency of sporogonic development is unlikely to be seen, or relevant; the mechanism of inhibition is unknown.

Several hypotheses may account for the observations that sporozoite prevalence was higher in *An. arabiensis* (Ifakara) that consumed cow rather than human blood, and was higher in *An. gambiae s.s.* (Keele) consuming human rather than cow blood. Firstly, this phenomenon could be a by-product of the co-evolutionary relationship between mosquitoes and hosts, from which parasites indirectly benefit. Mosquitoes may have evolved mechanisms for more efficient extraction of nutritional resources from blood of their preferred host species, which are then available for enhanced parasite growth. For example, *An. stephensi* mosquitoes are more efficient at concentrating erythrocytes (and haemoglobin) from the blood of their preferred bovine host than from human or mouse blood, and may thus obtain higher nutritional value from this source^39^,^40^. Mosquitoes may also digest blood meals from naturally preferred host species at a faster rate than other host types^41^, which could indirectly influence the nutritional value of the blood meal and/or parasite invasion kinetics. For example, a faster rate of digestion would destroy more ookinetes before they are able to leave the mosquito stomach; only ookinetes leaving fast would survive. Alternatively, the effectiveness with which mosquitoes mount effective anti-parasitic immune responses may be influenced by the source of blood they consume.

It is unclear why sporozoite numbers in *An. gambiae s.s.* (Keele) were lower after consumption of cow blood during sporogony compared to scenarios where they were given human blood or even no blood meal. One possibility is that cow blood provides a poor nutritional source for parasite development, or that it maximises the mosquito immune response to the sporozoite stages when travelling through the haemolymph to the salivary glands.

The observation of increased oocyst numbers in *An. gambiae s.s.* (Keele) mosquitoes consuming cow or human blood during sporogony, compared to unfed control mosquitoes (Fig. 2d), was unexpected. Oocysts establish 24–48 h after infection, earlier than the second blood meal was offered, and ookinetes that fail to cross the mosquito midgut wall during this period are expelled with the remains of the blood meal. The most likely explanation was an unusually high mortality observed in these experiments in the unfed control group compared to the two groups with additional blood feeding (Supplementary Table S2); the cause of this higher mortality is not known.

The findings reported here generate new hypotheses regarding the potential epidemiological consequences of shifts in malaria vector behaviour and species composition. Overall, a shift in vector species composition from highly anthropophilic species such as *An. gambiae s.s.* to more zoophilic species like *An. arabiensis* is expected to decrease transmission by reducing the human biting rate. Here we demonstrate, for the colonies used, that *An. arabiensis* mosquitoes fed *P. falciparum* gametocyte-infected blood are half as likely to produce infective sporozoites as *An. gambiae s.s.* feeding on the same blood. However, this reduction in the potential for transmission is ablated if *An. arabiensis* subsequently feed on their preferred bovine hosts.

The mosquito-parasite combination used here is naturally occurring, and the mosquito lines were chosen on the basis of having relatively high genetic diversity (*An. gambiae s.s.* (Keele)^38^), and being recently colonized from a field population (*An. arabiensis* (Ifakara)^42^), although it is inevitable that they have undergone adaptation to laboratory conditions. The results obtained cannot be directly extrapolated to natural settings without more extensive validation under field conditions. If the results described here are confirmed in wild vector populations, the magnitude of host feeding-related effects could be dampened or enhanced by other aspects of their ecology. Whereas the impact of the blood of different host species was tested under simplified conditions where infected mosquitoes had access to only one blood meal during sporogony, in the wild mosquitoes will blood-feed 3–6 times in this period^31^. The frequency and variety of blood sources consumed then could have multiple impacts on sporogony and mosquito survival that are not obvious under laboratory conditions. Additionally, the potential effects of mosquito diet on parasite sporogony will depend upon the range of host species available to mosquitoes, and on which species they choose to feed. By providing proof of principle that mosquito host choice can significantly alter the outcome of human malaria parasite sporogony in important African vector species, this study provides impetus for further investigation under more natural field conditions. The underlying mechanism behind the effect of blood source on sporogony, either via nutritional benefits to the parasite, or through altered mosquito immune responses, will be a key area for future studies.

Our results tentatively suggest that the benefits of eliminating highly anthropophilic species may be less than expected, if the more zoophilic feeding behaviour in the less permissive vectors that remain endow them with an equal probability of developing sporozoite stage infections, a view supported by the similar sporozoite prevalences observed in wild-caught and naturally infected *An. arabiensis, An. coluzzii* and *An. gambiae s.s*.^43^. Combined with other known facets of their behaviour that reduce their susceptibility to conventional control measures, e.g. ability to bite outdoors and early in the evening when people are not protected by bednets, the transmission potential of even small, residual populations of *An. arabiensis* could be considerable.

## Methods

### Mosquito rearing

Laboratory colonies of *An. gambiae s.s.* (Keele line)^38^ and *An. arabiensis* (Ifakara line)^42^ were reared under standard insectary conditions of 27 ± 1 °C, 70% humidity and a 12 h light: 12 h dark cycle. Larvae were fed *ad libitum* on TetraMin fish flakes (Tetra Ltd., UK). Adult mosquitoes were fed *ad libitum* on a 5% glucose solution supplemented with 0.05% (w/v) 4-aminobenzoic acid (PABA). Further details of the colonies are provided in the Supplementary Information.

### History of parasite and mosquito lines

Mosquitoes used in this study were from laboratory colonies of *An. arabiensis* (Ifakara line) and *An. gambiae s.s.* (Keele line). The *An. arabiensis* (Ifakara) colony was established in 2008 with individuals from Sagamaganga village (approx. 15 km from Ifakara Health Institute, Tanzania)^42^. The Keele colony was produced by balanced interbreeding of four *An. gambiae s.l.* strains (KIL from Marangu, Tanzania; G3 from MacCarthy Island, Gambia; ZAN U from Zanzibar, and Ifakara strain from Njage, Tanzania)^38^, and a colony was established in Glasgow in 2005. Both colonies have been maintained using membrane feeding with human blood in the Glasgow University insectaries. The Keele line has mixed ancestry with both M (now denoted *An. coluzzii*) and S (now denoted *An. gambiae s.s.*) genetic background; under laboratory conditions these interbreed to form fertile hybrids. Details of the molecular analysis of the Keele colony to characterise its species composition will be published elsewhere (Ranford-Cartwright LC *et al*., manuscript accepted for publication, Plos One).

*Plasmodium falciparum* clone 3D7 was derived from Dutch isolate NF54 by limiting dilution^44^. The origin of NF54 strain is reported in Delemarre and Van der Kaay (1979)^45^.

### Parasite culture and mosquito infections

Human blood and serum for parasite culture was obtained from the Glasgow and West of Scotland Blood Transfusion Service. Ethical approval for use of human blood and serum was obtained from the Scottish National Blood Transfusion Service Committee for Governance of Blood and Tissue Samples for Non-therapeutic Use. Whole blood from donors (any blood group) was washed before use to remove white blood cells, and resuspended in human serum (blood group AB, heat-inactivated). *P. falciparum* gametocyte cultures (clone 3D7) were set up according to standard procedures, with a mixture of gametocytes from a 14- and a 17- day old gametocyte culture used for each infectious feed, as described^46^. Gametocytes were diluted with uninfected blood to achieve approximately 0.7% gametocytaemia, which is known to generate an acceptable infection prevalence (>50%) in mosquitoes (L. Ranford-Cartwright, unpublished). Groups of ~240 female *An. gambiae s.s.* (Keele) and *An. arabiensis* (Ifakara) mosquitoes were fed on gametocyte-infected blood using membrane feeders as previously described^46^. Mosquitoes were allowed to feed for 15–20 minutes. Fully-fed mosquitoes were randomly allocated to one of three experimental treatments for a second blood meal (approximately 70–80 individuals per treatment): (1) human blood, (2) cow blood, or (3) no blood (control group). These groups of mosquitoes were maintained under standard insectary conditions with access to a solution of 5% glucose containing 0.05% PABA. The second uninfected blood meal of either human or cow blood was offered to mosquitoes via membrane feeders four days after the infectious blood meal, with the control group receiving nothing. Four days was chosen based on the known feeding patterns of these mosquitoes species in nature^32^. Human and cow blood for the second mosquito blood feeds was obtained from a commercial source (Patricell, BioCity Nottingham, UK) and used without further washing steps. Mosquitoes that did not take a second blood meal when offered were removed. The entire experiment was repeated three times for each mosquito species, generating a total sample size of approximately 240 mosquitoes in each group.

### Measuring mosquito fitness and infection prevalence

From the day after the second blood meal onwards, mosquito-holding pots were examined daily to record any mortality. On day 10 after the initial infectious feed, a random sub-sample of mosquitoes from each treatment and vector species (20–30) was killed to assess infection by midgut examination. The number of oocysts on the gut was counted (400X magnification), and then the whole midgut was preserved for DNA analysis by incubation overnight at 55 °C in 500 μl of lysis buffer containing 1 mg/ml Proteinase K, and then stored at −80 °C until DNA extraction^47^. A standard indicator of body size (winglength: the distance from the axillary incision to the apical margin)^48^,^49^,^50^ was measured using a digital camera imaging system (Moticam 2300, 20x magnification), using pre-calibrated software (Motic Images Plus, v.2.0). The amount of blood taken has been shown to correlate with mosquito body size^51^,^52^. A similar process was performed on days 15–16 after the infectious feed to measure the presence and abundance of sporozoites within mosquito salivary glands. Salivary glands from individual mosquitoes were processed as described for midguts. When all six lobes of the salivary glands were not obtained, the number of complete lobes that were isolated was recorded and used as an additional covariate in subsequent statistical analysis.

### Quantitative real-time polymerase chain reaction (qPCR) assay

Quantitative real-time PCR (qPCR) has been applied to estimate malaria parasite abundance in mosquitoes^53^, allowing quantification of the numbers of parasite genomes present within individual oocysts, within all oocysts on mosquito midguts, or within salivary glands, and can be interpreted as the number of sporozoites developing within them^53^. Reactions were performed on a Roche Light Cycler using SYBR Green. DNA standards containing known numbers of *P. falciparum* parasites were produced from asexual cultures (following^53^), and used to generate standard curves of *P. falciparum* as previously described^53^. Further details of the methodology are given in the Supplementary Information.

### Statistical analysis

The influence of blood type on the presence and abundance of parasites developing in mosquito midguts and salivary glands was analysed statistically. Five measures of parasite sporogonic progress were assessed: (1) the proportion of midguts infected with oocysts (oocyst prevalence), (2) the number of oocysts per midgut (oocyst load), (3) the number of parasite genomes per midgut, (4) the proportion of salivary glands infected with sporozoites (sporozoite prevalence), and (5) the number of sporozoites present in mosquito salivary glands. In practice, sporozoite prevalence gives the most direct estimate of parasite transmission potential to new hosts as only mosquitoes infected with sporozoites can transmit, and abundance is unlikely to be a limiting factor because very low numbers are needed to start new infections (>25^54^,^55^), whereas a single oocyst can give rise to hundreds or thousands of sporozoites^56^,^57^. In all analyses, host blood species and mosquito species were investigated as the main effects of interest. Mosquito body size was fitted as an additional fixed explanatory variable, due to its considerable influence on mosquito feeding and fitness parameters^51^,^52^. Generalised Linear Mixed Models (GLMM) assuming a binomial distribution were used to test the relationship between mosquito species and host blood species on the binary response variables of oocyst and sporozoite prevalence (absent or present, lme4 package, R statistical software v2.12.2^58^). A maximal model was constructed that included all fixed effects and their interactions plus the random effect of experimental replicate. Backward elimination was used for sequential removal of non-significant variables, to obtain the minimal statistically-significant model^59^. A similar approach was used to test for variation in the number of parasites (oocyst load, sporozoites per midgut, and total number of sporozoites in salivary glands). Given the highly over-dispersed nature of parasite abundance data, a negative binomial distribution was assumed in these GLMMs (glmmADMB, nlme package, R software^58^). A backwards elimination approach was used to test for the significance of all fixed effects (mosquito species, host blood species, wing length) and their interactions, while controlling for random variation due to replicate, as was performed for the analyses of oocyst and sporozoite prevalence. In analysing variation in the total number of sporozoites per midgut, the additional explanatory variable of oocyst load (number of oocysts per midgut) was included. Oocyst load was classified into a two-level categorical variable of low (<10 oocysts) and high intensity (≥10 oocysts per midgut). This categorization was justified on the basis of natural break points in the distribution of oocysts within infected mosquitoes in these experiments, and also biological significance under natural settings, as the low intensity group is typical of oocyst infection intensities observed in the field^60^. The explanatory variable of number of complete salivary gland lobes was also included in the analysis of the total number of sporozoites in salivary glands.

## Additional Information

**How to cite this article**: Emami, S. N. *et al*. The transmission potential of malaria-infected mosquitoes (*An.gambiae*-Keele, *An.arabiensis*-Ifakara) is altered by the vertebrate blood type they consume during parasite development. *Sci. Rep.* **7**, 40520; doi: 10.1038/srep40520 (2017).

**Publisher's note:** Springer Nature remains neutral with regard to jurisdictional claims in published maps and institutional affiliations.

## Supplementary Material

Supplementary Information
