# Supplementary material for: The transmission potential of malaria-infected mosquitoes (An.gambiae-Keele, An.arabiensis-Ifakara) is altered by the vertebrate blood type they consume during parasite development
Source: Sci Rep. 2017 Jan 17;7:40520. doi: 10.1038/srep40520 (PMC5240107; doi:10.1038/srep40520)
Supplement: Supplementary Information [file srep40520-s1.pdf]

The transmission potential of malaria-infected mosquitoes (*An.gambiae*-Keele, *An.arabiensis*-Ifakara) is altered by the vertebrate blood type they consume during parasite development

S. Noushin Emami, Lisa C. Ranford-Cartwright, Heather M. Ferguson

Supplementary Information

Supplementary Table 1. Mosquito sample sizes.

Species (line)	Source of blood 4 days after infection	# Receiving a *P. falciparum* infected blood meal[a]	# Dissected at day 10 (oocyst stage)	# Dissected at day 15-16 (sporozoite stage)[b]
***An. arabiensis* (Ifakara)**	Human	80 × 3	71	53
	Cow	80 × 3	62	70
	Control	80 × 3	60	43
	Total	720	193	166
***An. gambiae s.s.* (Keele)**	Human	80 × 3	68	70
	Cow	80 × 3	66	76
	Control	80 × 3	60	69
	Total	720	194	215

[a] All mosquitoes that were offered a blood meal took one, i.e. there were no unfed mosquitoes.
[b] Not all of the remaining mosquitoes at D16 were dissected for sporozoite infection.

Supplementary Table 2. Mosquito deaths occurring between day 4 and day 10 post infection. The number of mosquito deaths occurring between day 4 (when the second blood meal treatment was offered) and day 10 (when ~half the survivors were dissected to check for oocysts), and between day 10 (remaining after oocyst dissection) and day 16, are presented for each replicate separately. The rate of mortality between day 4 and 10 was significantly lower among mosquitoes taking a second blood meal compared with the control group that did not receive a second blood meal [cow/control: z=5.02, p<0.001; human/control: z=5.58, p<0.001]. The source of second blood meal had no significant effect on mosquito death rates [death (4-10): cow/human: z=-1.04, p=0.55; death (10-16): cow/human: z=-1.62, p=0.23]. Death rate was not significantly influenced by mosquito species [death (4-10):F=0.83, p=0.67; death (10-16): F=0.33, p=0.57] or by its interaction with the second blood meal treatment [mosquito species * host blood treatment: death (4-10): χ^2=2.73, p=0.25; death (10-16): χ^2=2.18, p=0.14]. All analyses were performed using GLMM models.

**Species (line)**	**Second blood meal treatment (given on day 4)**	**Replicate**	**Number of mosquitoes that received a second blood meal treatment**	**Number of mosquitoes dying between day 4 - 10**	**Number of mosquitoes dying between day 10 - 16**
***An. arabiensis* (Ifakara)**	Human	1	80	0	8
	Cow	1	79	3	9
	Control	1	70	6	13
***An. gambiae s.s.* (Keele)**	Human	1	78	2	7
	Cow	1	77	5	9
	Control	1	77	18	10
***An. arabiensis* (Ifakara)**	Human	2	77	3	5
	Cow	2	78	4	4
	Control	2	70	14	3
***An. gambiae s.s.* (Keele)**	Human	2	78	3	5
	Cow	2	78	3	9
	Control	2	75	10	4
***An. arabiensis* (Ifakara)**	Human	3	77	4	2
	Cow	3	78	3	4
	Control	3	70	19	4
***An. gambiae s.s.* (Keele)**	Human	3	78	10	0
	Cow	3	78	10	3
	Control	3	75	15	0

Supplementary Table 3. Median of parasite numbers within *An. gambiae s.s.* (Keele) and *An. arabiensis* (Ifakara) mosquitoes taking a second non-infectious blood meal on cow or human blood, or no second blood meal (control). Numbers in brackets represent 95% confidence intervals. The data are shown for infected mosquitoes only (Inf) or for total mosquitoes (Total). Data are pooled from the three replicates.

		***An. arabiensis* (Ifakara)**			***An. gambiae* s.s. (Keele)**		
		**Control**	**Cow**	**Human**	**Control**	**Cow**	**Human**
**Oocysts per midgut**	**n (Inf/Total) [a]**	27/37	31/35	28/37	17/38	28/41	24/38
	**Inf: median (95%CI) [b]**	6 (2-12)	13 (4-24)	6 (3-14)	3 (1-6)	5 (3-16)	15 (2-30)
	**Total: median (95%CI) [c]**	3 (0-11)	12 (3-20)	3 (0-10)	1 (0-3)	3 (0-8)	2 (0-7)
**Parasite genomes / midgut**	**n (Inf/Total) [a]**	27/37	31/35	28/37	17/38	28/41	24/38
	**Inf: median (95%CI) [b]**	1030 (143-1510)	515 (218-1660)	201 (79-508)	5036 (3274-15033)	5048 (768-50013)	3013 (1016-17521)
	**Total: median (95%CI) [c]**	5 (0-835)	502 (175-1538)	114 (3-438)	2280 (0-6287)	2007 (221-47514)	2013 (357-5025)
**Parasite genomes / salivary glands**	**n (Inf/Total) [a]**	24/63	48/66	29/66	44/63	39/96	48/66
	**Inf: median (95%CI) [b]**	415 (343-898)	1016 (298-4646)	1526 (525-5024)	510 (253-2012)	25018 (4262-50035)	20006 (5008- 31275)
	**Total: median (95%CI) [c]**	0 (0-5003)	314 (0-2040)	0 (0-659)	302 (0-1029)	151 (0-25023)	5038 (137-25045)

[a] n (Inf/total)= total number of infected mosquitoes / total sample
[b] Inf: median(95%CI)= median of infected mosquitoes only (95% confidence interval)
[c] total: median(95%CI)= median of infected and non-infected mosquitoes (95% confidence interval)

Supplementary Table 4. Median oocyst load of *P. falciparum* within *An. gambiae s.s.* (Keele) and *An. arabiensis* (Ifakara) mosquitoes taking a second non-infectious blood meal on cow or human blood, or no second blood meal (control), by replicate. Numbers in brackets represent range. Data show infected mosquitoes only.

	Second blood meal origin	Replicate 1	Replicate 2	Replicate 3
***An. arabiensis* (Ifakara)**	Control	3 (1-40)	6 (5-39)	6 (1-30)
	Cow	25 (3-40)	12 (3-45)	13 (3-42)
	Human	5 (1-46)	4 (1-61)	4 (1-50)
***An. gambiae s.s.* (Keele)**	Control	3 (1-7)	4 (1-7)	3 (1-8)
	Cow	5 (1-21)	17 (3-83)	15 (2-56)
	Human	2 (1-15)	26 (2-85)	20 (2-75)

Supplementary Table 5. Summary of negative binomial parameters for estimating the efficiency of parasite development (parasite load) from midgut to salivary gland infection.

Mosquito species	Infection site	Source of blood meal 4 days after infection	Beta estimation (negative binomial model)
***An. arabiensis* (Ifakara)**	Salivary gland	control	6.88
		cow	7.89
		human	9.36
***An. gambiae s.s.* (Keele)**	Salivary gland	control	7.84
		cow	10.67
		human	10.24
***An. arabiensis* (Ifakara)**	Midgut	control	7.14
		cow	7.25
		human	6.47
***An. gambiae s.s.* (Keele)**	Midgut	control	9.57
		cow	9.91
		human	9.21

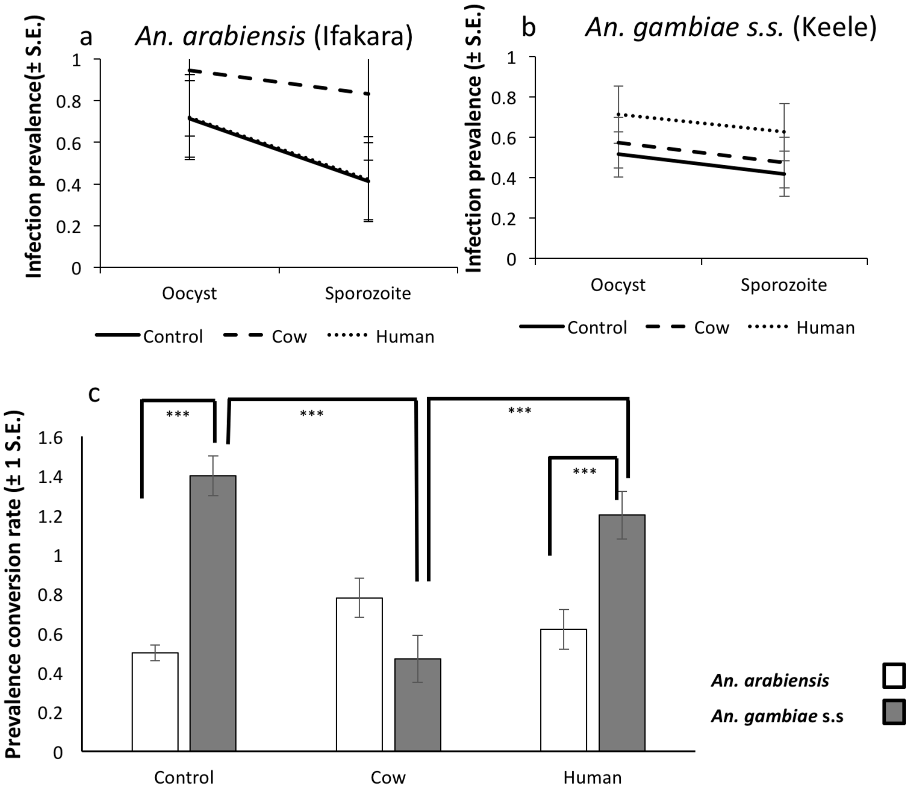

Supplementary Figure 1. Infection rates in (a) *An. arabiensis* (Ifakara) and (b) *An. gambiae s.s.* (Keele) estimated from the glmm comparing oocyst and sporozoite infection location. The effect of host blood type and mosquito species, and their interaction, on conversion efficiency, was analysed using glmer. The "slope" of the infection stage*blood meal interaction was estimated; values not statistically different from 1 represented 100% conversion, whereas a slope significantly more or less than 1 indicated a variation in conversion efficiency on the basis of host blood type. Because of a significant 3-way interaction (infection stage*blood type*mosquito species), separate analyses were performed for each mosquito species, in which the significance of the infection stage*blood type was estimated. Error bars are standard errors from the model. (c) Conversion rate from midgut oocyst to salivary gland sporozoite positivity was estimated using glmm (glmer) analysis. Estimates of sporozoite and oocyst prevalence from this model were used to calculate "infection conversion efficiency" as their quotient (sporozoite prevalence/ oocyst prevalence), with values of 1 indicating no change in infection prevalence between midgut and salivary stages. The error bars are standard errors. White bars show *An. arabiensis* (Ifakara), and gray bars are for *An. gambiae s.s.* (Keele). Statistically different comparisons are shown by the brackets (***p≤0.001; *p=0.01).

Supplementary methodology

Estimation of sporozoite numbers by Quantitative PCR using *Pfg377* gene.

For the generation of a standard curve of known numbers of parasites, separate 100 µL samples containing 1.25, 2.5, 5, 10, 100, 1000, 1 x 10^4, and 1 x 10^5 asexual parasites per microlitre of culture were prepared by dilution in uninfected blood at 5% haematocrit. To

estimate the total number of parasites per microlitre of culture, values of parasitaemia were multiplied by red blood cell density of cultures.

DNA was extracted from mosquito and culture samples (for the standard curve) using a standard protocol (Charge Switch®gDNA micro tissue kit, Invitrogen, UK, CS 11203) following the manufacturer's protocols. The DNA was eluted from the beads with 100 µL of elution buffer (E5, 10mM Tris-HCl, pH 8.5), and the purified DNA samples stored at -80°C.

The quantitative reactions were prepared in Lightcycler glass capillary reaction vessels in a final volume of 20 µL, containing 600nM of each primer (377F: 5' ACTCCAGAAGAAGAAGAGCAAGC3'; 377R:5'TTCATCAGTAAAAAAAGAATCGTCATC 3'), 3mM magnesium chloride, and 1 x Roche Light Cycler Fast Start DNA Master SYBR Green I hot start reaction mix containing with 4 µL of sample template DNA. Each sample was run in duplicate within a single run. Standard curves were generated for every quantitative real time PCR assay run, using the prepared samples of suitable concentrations. Each assay included five DNA standards of 500, 1000, 2500, 5000, and 10000 parasites (per reaction), run in duplicate. DNA extracted from midguts and salivary glands taken from uninfected mosquitoes were used as negative controls.

The cycling profile comprised an initial denaturation (hot start) of 95°C for 600 s and then 35 amplification cycles of denaturation 95°C for 30 s, annealing 55°C for 20s and extension 65°C for 30s. Under these conditions the assay amplified only the 408 bp target amplicon with a characteristic melting temperature of 80.7°C. There was no amplification of human or mosquito DNA, or primer-dimer formation (data not shown).

Light Cycler data were analysed using the Fit Points method to obtain a (mean) genome number for each sample by comparison with the standard curve drawn from the DNA standards.
